# Adenosine stress CMR T1-mapping detects early microvascular dysfunction in patients with type 2 diabetes mellitus without obstructive coronary artery disease

**DOI:** 10.1186/s12968-017-0397-8

**Published:** 2017-10-25

**Authors:** Eylem Levelt, Stefan K. Piechnik, Alexander Liu, Rohan S. Wijesurendra, Masliza Mahmod, Rina Ariga, Jane M. Francis, Andreas Greiser, Kieran Clarke, Stefan Neubauer, Vanessa M. Ferreira, Theodoros D. Karamitsos

**Affiliations:** 1 University of Oxford Centre for Clinical Magnetic Resonance Research, Division of Cardiovascular Medicine, Radcliffe Department of Medicine, University of Oxford, Oxford, UK; 2 Department of Cardiovascular Sciences, University of Leicester, Leicester, UK; 3 Siemens Healthcare GmbH, Erlangen, Germany; 4 Department of Physiology, Anatomy and Genetics, University of Oxford, Oxford, UK; 5 1st Department of Cardiology, Aristotle University of Thessaloniki, AHEPA Hospital St. Kyriakidi 1, 54636 Thessaloniki, Greece

**Keywords:** Cardiovascular magnetic resonance, Diabetes mellitus, Myocardial perfusion, ShMOLLI T1-mapping, Microvascular obstruction

## Abstract

**Background:**

Type 2 diabetes mellitus (T2DM) is associated with coronary microvascular dysfunction in the absence of obstructive coronary artery disease (CAD). Cardiovascular magnetic resonance (CMR) T1-mapping at rest and during adenosine stress can assess coronary vascular reactivity. We hypothesised that the non-contrast T1 response to vasodilator stress will be altered in patients with T2DM without CAD compared to controls due to coronary microvascular dysfunction.

**Methods:**

Thirty-one patients with T2DM and sixteen matched healthy controls underwent CMR (3 T) for cine, rest and adenosine stress non-contrast T1-mapping (ShMOLLI), first-pass perfusion and late gadolinium enhancement (LGE) imaging. Significant CAD (>50% coronary luminal stenosis) was excluded in all patients by coronary computed tomographic angiography.

**Results:**

All subjects had normal left ventricular (LV) ejection and LV mass index, with no LGE. Myocardial perfusion reserve index (MPRI) was lower in T2DM than in controls (1.60 ± 0.44 vs 2.01 ± 0.42; *p* = 0.008). There was no difference in rest native T1 values (*p* = 0.59). During adenosine stress, T1 values increased significantly in both T2DM patients (from 1196 ± 32 ms to 1244 ± 44 ms, *p* < 0.001) and controls (from 1194 ± 26 ms to 1273 ± 44 ms, p < 0.001). T2DM patients showed blunted relative stress non-contrast T1 response (T2DM: ΔT1 = 4.1 ± 2.9% vs. controls: ΔT1 = 6.6 ± 2.6%, *p* = 0.007) due to a blunted maximal T1 during adenosine stress (T2DM 1244 ± 44 ms vs. controls 1273 ± 44 ms, *p* = 0.045).

**Conclusions:**

Patients with well controlled T2DM, even in the absence of arterial hypertension and significant CAD, exhibit blunted maximal non-contrast T1 response during adenosine vasodilatory stress, likely reflecting coronary microvascular dysfunction. Adenosine stress and rest T1 mapping can detect subclinical abnormalities of the coronary microvasculature, without the need for gadolinium contrast agents. CMR may identify early features of the diabetic heart phenotype and subclinical cardiac risk markers in patients with T2DM, providing an opportunity for early therapeutic intervention.

## Background

Type 2 diabetes mellitus (T2DM), independent of coronary artery disease (CAD) or hypertension, directly affects the structure and function of the myocardium [1], leading to adverse cardiovascular outcomes [2–4]. There is a robust association between T2DM and heart failure, and heart failure is the leading cause of mortality in patients with T2DM [5–8]. Coronary microvascular dysfunction in diabetes is a complex pathophysiological process, which involves structural, functional and metabolic alterations, and has emerged among the potential mechanisms leading to increased incidence of heart failure [9] and risk of cardiovascular mortality [4, 10] in patients with diabetes. Using cardiovascular magnetic resonance (CMR), myocardial perfusion during vasodilator stress has been shown to be impaired in patients with diabetes mellitus (DM) [11–13] and in the absence of epicardial coronary artery stenoses, this finding is indicative of coronary microvascular dysfunction.

CMR non-contrast T1-mapping may be used to assess myocardial ischaemia and coronary vasodilatory function. In CMR, T1 (or proton spin-lattice) relaxation time is a magnetic property of tissue. Each tissue type has a normal range of T1 values, deviation from each may indicate disease or a change in physiology. T1 is prolonged by increased free water content [14], which may have an intracellular or extracellular origin, including the interstitial and intravascular compartments. Healthy controls demonstrate normal myocardial T1 values at rest; during adenosine stress, coronary vasodilation leads to an increase in myocardial blood volume in the myocardium, which is detectable using myocardial T1-mapping, through the partial volume of blood T1 [14–16]. We have previously demonstrated the use of adenosine stress and rest non-contrast T1-mapping for detecting ischaemia in patients with CAD, and for assessing coronary vasodilatory reserve in patients without CAD [17, 18]. In the absence of obstructive CAD, the percentage change in non-contrast T1 values from rest to vasodilator stress (ΔT1) likely represents coronary vascular reactivity and has potential to assess the health and function of the micro coronary circulation.

We hypothesize that patients with T2DM but without obstructive CAD will have microvascular dysfunction detectable using adenosine stress and rest T1-mapping. In this study, we compared native myocardial T1 values at rest and during adenosine stress, as well as myocardial perfusion reserve index (MPRI) in patients with T2DM without obstructive CAD and in matching healthy controls using CMR at 3 Tesla.

## Methods

The study was granted a favourable opinion by the National Research Ethics Committee (REC Ref 13/SW/0257), and written informed consent was obtained from each participant. The main project was set out to investigate the intricate interplay of perfusion, oxygenation and metabolic changes during stress in the diabetic heart. The wider aims and methods of the main project has been published elsewhere [13].

### Study population

T2DM was diagnosed according to the World Health Organization criteria [19]. Patients with T2DM were excluded if they had a history of cardiovascular disease, chest pain, tobacco smoking, uncontrolled hypertension (resting systolic blood pressure (BP) >140 mmHg and diastolic BP >90 mmHg), contraindications to CMR imaging, ischemic changes on 12-lead electrocardiogram (ECG), or significant renal impairment (estimated glomerular filtration rate below 30 mL/min), and if they were taking insulin. Importantly, patients with T2DM were initially screened for obstructive epicardial CAD (>50% of luminal stenosis) with coronary computed tomographic angiography (CCTA). Only subjects with no evidence of significant epicardial CAD on CCTA underwent the study investigations.

Healthy subjects were age-, gender- and weight-matched to patients and served as controls. Healthy controls had no history of heart disease, DM, hypertension, or high cholesterol and were taking no medications. They had normal physical examinations and ECG.

### Other study procedures

BP was recorded as an average of 3 supine measurements taken over 10 min (DINAMAP-1846-SX, Critikon Corporation, Tampa, Florida, USA). Fasting venous blood was drawn for glucose, triglyceride, haemoglobin A1c and renal function tests as previously described [20].

### Coronary computed tomographic angiography

CCTA scans were performed on a 64-slice CT scanner (Discovery CT 690, GE Healthcare, Waukesha, Wisconsin, USA) in accordance with performance guidelines from the Society of Cardiovascular Computed Tomography [21]. In the absence of contraindications, patients received beta-blockade (intravenous metoprolol) and sublingual nitroglycerine prior to the scan to achieve a heart rate of <65 beats per minute and coronary dilation, respectively. A preliminary unenhanced scan was performed to assess coronary artery calcium score. During the CCTA acquisition, 80 ml of iodinated contrast (Visipaque, GE Healthcare, Princeton, New Jersey, USA) was injected followed by a 50 ml saline flush. Significant CAD was defined as >50% luminal stenosis. CCTA was carried out 1 week prior to CMR study for each participant withT2DM.

### Cardiovascular magnetic resonance protocol

CMR was performed on a 3-Tesla system (Magnetom Trio Tim; Siemens Healthcare, Erlangen, Germany). All participants were instructed to refrain from caffeine ingestion in the 24 h preceding the study. All subjects were scanned after fasting overnight. Cine imaging was performed in long- and short-axis covering the left ventricle (LV) using standard balanced-steady state free precession (bSSFP) methods [22].

At rest, mid LV native T1 map was acquired using the shortened modified Look-Locker inversion recovery (ShMOLLI) prototype sequence as previously described [14], matched to the first-pass stress and rest perfusion mid-LV slices. Adenosine stress perfusion imaging was performed as previously described [23, 24] with acquisition of 3 matched short axis stress and rest perfusion images. Briefly, adenosine (140 μg/kg/min to 210 μg/kg/min to achieve adequate hemodynamic stress response) was infused intravenously for at least 3 min. Heart rate and blood pressure were recorded at baseline and at 1-min intervals during stress. This was followed by acquisition of a single mid-ventricular short-axis T1-map (matched to the mid-ventricular T1-map at rest). Subsequently (4–5 min after commencing the adenosine infusion), a 0.03-mmol/kg bolus of gadolinium-based contrast Gadoterate meglumine (Dotarem, Guerbet LLC, Villepinte, France) was injected, followed by 15 mL of normal saline at a rate of 6 mL/s for first-pass perfusion imaging. Adenosine was then discontinued, and after at least 20 min (to allow contrast washout adenosine), another 0.03-mmol/kg bolus of gadolinium was given for rest perfusion imaging.

For late gadolinium enhancement (LGE) CMR, a top-up bolus of 0.09 mmol/kg of contrast (Gadoteric acid, Guerbet, France) followed by a 15-mL saline flush was administered immediately after the rest perfusion image acquisition (total dose of gadolinium 0.15 mmol/kg). The LGE images were acquired using a T1-weighted phase-sensitive inversion recovery sequence 8–12 min after the last intravenous administration of contrast agent in the same long- and short-axis images as cine imaging, using imaging parameters as previously described [25]. A single mid-ventricular short-axis slice matching the pre-contrast T1-map was acquired 15 min after the administration of gadolinium contrast for extracellular volume fraction (ECV) quantification.

### CMR data analysis

For each patient, LV volumes, ejection fraction, and mass were calculated using cmr42© software (Circle Cardiovascular Imaging Inc., Calgary, Canada) by manually tracing the endocardial and epicardial contours in end-diastolic and end-systolic images, as previously described [22].

**Fig. 1:**
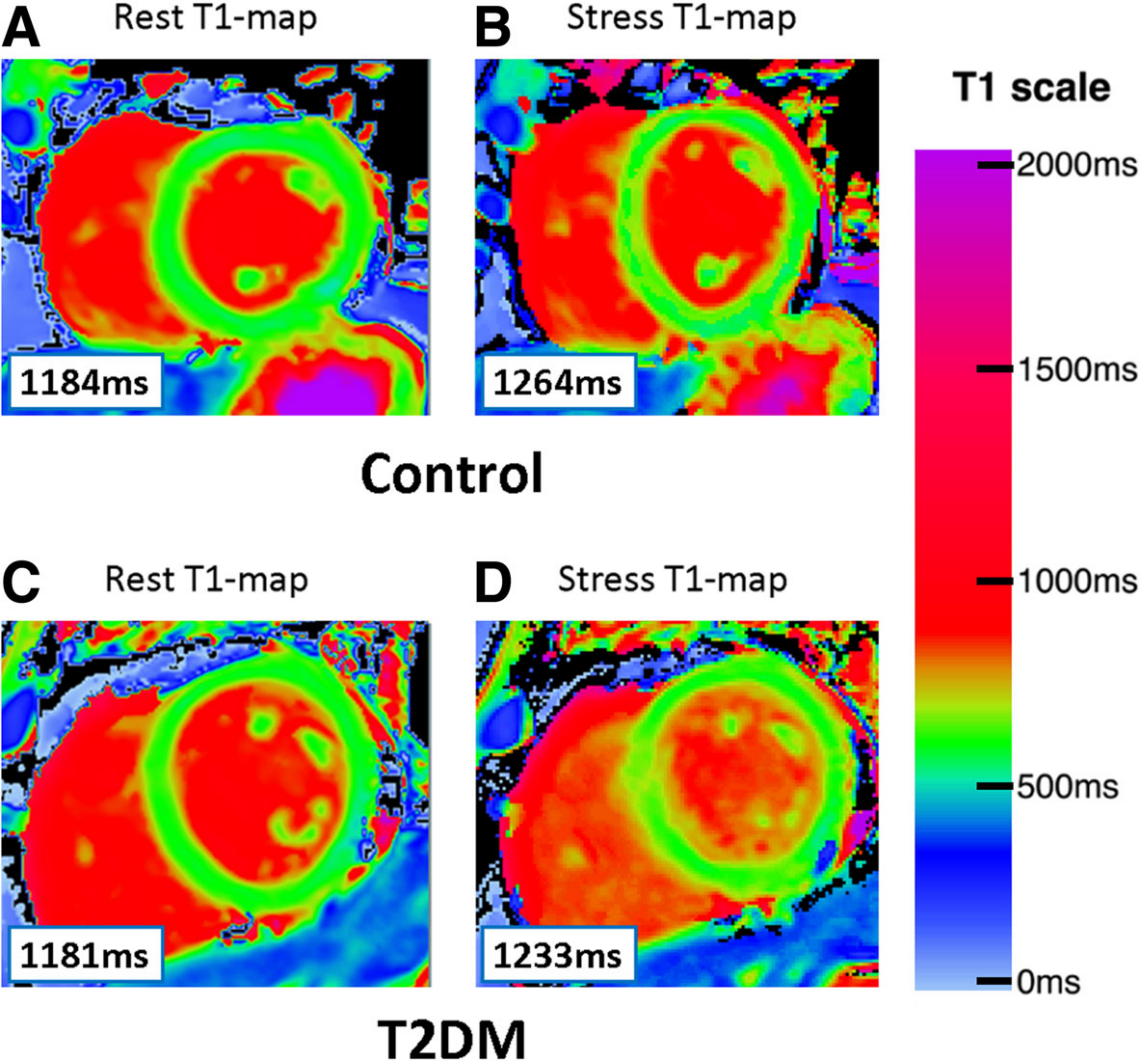
Representative examples of rest and stress T1 maps. Normal control with **a** resting native T1-map, 1174 ms; **b** corresponding stress T1-map, 1271 ms. T2DM patient with **c** resting native T1-map, 1177 ms; **d** corresponding stress T1-map, 1195 ms. Values in graphic indicate the average mid-left-ventricular myocardial T1 values

For the analysis of ShMOLLI T1-maps, the LV myocardium of the mid ventricular short axis slice acquired at baseline was contoured by two operators (EL, SKP), using dedicated software, as previously described [26], providing a single average myocardial T1 value from the whole slice of T1 map per each individual with consensus of two operators. T1-maps were assessed for quality in three ways: examination of the T1-map, the raw T1 images and R^2^ maps; ~11% of segments were excluded due to any combination of: off-resonance artifacts, partial-volume effects, poor T1 fit on the R^2^ maps, patient movement or low signal to noise, similar to previous publications using T1-mapping from our center [27]. The myocardial T1 value from the stress mid-ventricular ShMOLLI non-contrast T1-map was obtained and compared to the non-contrast T1 at baseline **(**Fig.1 **)**.

T1 reactivity to adenosine stress was expressed in absolute terms: ΔT1(ms) = T1stress–T1rest and as percentages: (ΔT1) (%) = ΔT1÷T1rest × 100. T1rest and T1stress represent mean T1 values at rest and during adenosine stress, respectively. Inter-observer variability of mid-ventricular ShMOLLI T1-map stress reactivity was assessed by further analysis performed by another independent operator in a blinded fashion (RSW). Inter-study variability of mid-ventricular ShMOLLI T1-map stress reactivity was assessed in 5 healthy subjects of another study performed at our research centre (REC reference: 13/SC/0376). These scans were anonymized and analyzed in random order in a blinded fashion (AL).

For analysis of myocardial perfusion, signal intensity over time curves were generated by tracing endocardial and epicardial contours (cmr42© software, Circle Cardiovascular Imaging Inc.) after manual correction for displacement during breathing, by two operators (AL, EL). A region of interest was drawn in the LV blood pool, avoiding any papillary muscles, to permit the derivation of an arterial input function. The myocardium was divided into equiangular segments on the basis of the American Heart Association 16-segmentation model. Rest and stress myocardial perfusion up-slopes were calculated using a 5-point linear-fit model of SI versus time and normalized to the LV blood pool upslope. Myocardial perfusion reserve index (MPRI) was defined as the ratio of stress to rest relative upslope, as previously described [28].

For LGE analysis, areas of contrast enhancement were visually scored as absent or present by 2 operators with at least 3 years of CMR experience (EL, MM). LGE was considered present only if myocardial enhancement was confirmed on both short-axis and perpendicular long-axis locations. Consistent with established methods of estimating myocardial ECV using a delayed postcontrast bolus protocol [29, 30], we measured precontrast and postcontrast myocardial and blood T1 values. These assessments were carried out by two operators together by consensus (EL, SKP), using previously described methods [30].

### Statistical analysis

All data are expressed as mean ± standard deviations and checked for normality using the Kolmogorov–Smirnov test. Categorical data are presented as numbers and percentages. Comparisons between the 2 groups were performed by Student’s t-test. The chi-square test or Fisher’s exact test was used to compare discrete data as appropriate. Bivariate correlations were performed using Pearson’s or Spearman’s method as appropriate. A *p*-value <0.05 was considered significant. All statistical analyses were performed with SPSS Statistics (version 20; International Business Machines, Inc., Armonk, New York, USA).

## Results

### Participant characteristics

Demographic, clinical, and biochemical data of patients with T2DM and controls matched for age, gender and body mass index (BMI) are shown in Table 1. 31 patients with T2DM (median duration of diabetes 7 years [IQR: 1–8] and mean glycated haemoglobin level 7.7 ± 1.4%) and 16 controls were studied. Although resting heart rates were statistically higher in patients, these remained within the normal range (T2DM resting heart rate: 69 ± 8 vs. controls: 58 ± 10 beats per minute, Table 1). Diastolic BP and resting heart rate were statistically higher in the diabetic cohort, although remained within the normal range. A significant proportion of patients (77%) were on statin therapy, hence total cholesterol and low-density lipoprotein cholesterol levels were lower in diabetics compared to controls.

**Table 1:** Baseline characteristics of the study cohort

Variable	Healthy Controls *N* = 16	T2DM *N* = 31	*P* value
Age, y	51 ± 9	55 ± 9	0.162
BMI, kg/m^2^	25.8 ± 4.2	28.7 ± 5.6	0.083
Male, %	53	58	0.739
Diabetes duration, y	…	7[IQR:1–8]	
Systolic blood pressure, mmHg	121 ± 13	125 ± 11	0.366
Diastolic blood pressure, mmHg	71 ± 9	77 ± 8	0.048
Resting heart rate, bpm	58 ± 10	69 ± 8	<0.001
Plasma fasting glucose	4.8 ± 0.5	9.1 ± 3.2	<0.001
Plasma triglycerides, mmol/L	1.25 ± 0.68	1.47 ± 0.71	0.391
Total cholesterol, mmol/L	3.58 ± 0.93	1.99 ± 0.63	<0.001
HDL, mmol/L	1.32 ± 0.37	1.22 ± 0.36	0.442
LDL, mmol/L	2.86 ± 0.56	1.99 ± 0.63	<0.001
Hematocrit, %	41 ± 4	42 ± 3	0.501
Medications, n (%)			
Metformin	–	30 (97)	
Sulphonylurea	–	21 (68)	
Aspirin	–	11 (35)	
Statin	–	24 (77)	
ACE-I	–	21 (68)	

Values are mean ± standard deviations or percentages. T2DM indicates type 2 diabetes mellitus; BMI, body mass index; y, years; bpm, beats per minute; HDL, high density lipoprotein; LDL, low density lipoprotein; ACE-I angiotensin-converting enzyme inhibitors

### Myocardial structure and systolic function

CMR results for LV volumes and function are summarized in Table 2. There was no significant difference in LV ejection fraction between patients with T2DM and healthy controls. There was also no difference in LV mass and LV mass index between patients and healthy controls, though patients with T2DM exhibited concentric LV remodelling (LV mass to volume ratio T2DM: 0.96 ± 0.18 g/ml vs. healthy controls: 0.75 ± 0.23 g/ml; *p* = 0.001).

**Table 2:** Cardiovascular magnetic resonance results in patients vs. controls

Variable	Healhty Controls *N* = 16	T2DM *N* = 31	*P* value
LV end-diastolic volume, ml	159 ± 33	128 ± 33	0.004
Indexed LV end-diastolic volume, ml/m^2^	78 ± 19	64 ± 21	0.012
LV end-systolic volume, ml	49 ± 12	40 ± 17	0.087
Indexed LV end-systolic volume, ml/m^2^	23 ± 13	20 ± 16	0.168
LV stroke volume, ml	110 ± 22	87 ± 23	0.002
Indexed LV stroke volume, ml/m^2^	55 ± 25	45 ± 20	0.008
LV ejection fraction, %	69 ± 4	69 ± 8	0.786
LV mass index, g/m^2^	53 ± 15	60 ± 13	0.075
LV mass, g	115 ± 29	120 ± 31	0.534
LV mass/end-diastolic volume g/ml	0.75 ± 0.23	0.96 ± 0.18	0.001

Values are mean ± standard deviations or percentages. T2DM indicates type 2 diabetes mellitus; CMR, cardiovascular magnetic resonance; LV, left-ventricular

### Myocardial fibrosis on late gadolinium enhancement imaging

On visual assessment of LGE images, there were no areas of myocardial enhancement indicative of myocardial infarction or focal replacement fibrosis in either patients or healthy controls.

### Changes in myocardial perfusion reserve during adenosine stress

Rest and stress rate pressure product values are summarised in Table 3. Patients and healthy controls demonstrated a similar increase in rate pressure product during adenosine stress (*p* = 0.206). Despite the absence of significant obstructive epicardial CAD on CCTA and adequate response to adenosine vasodilatory stress, MPRI was significantly reduced in T2DM patients compared to controls (T2DM MPRI, 1.60 ± 0.44 vs healthy controls, 2.01 ± 0.42; *p* = 0.008). A sub-group analysis of lean T2DM patients (BMI < 25) vs lean controls for the MPRI was performed and our results showed in the absence of obesity or even overweight status T2D is associated with reduced MPRI (1.50 ± 0.27 vs lean controls 2.24 ± 0.36, *p* = 0.005).

**Table 3:** Haemodynamic measurements and myocardial perfusion reserve index

Adenosine Stress CMR			
	Healthy Controls *N* = 16	T2DM *N* = 31	*P* value
Rest RPP, bpm*mmHg	7673 ± 1334	8862 ± 1745	0.025
Stress RPP, bpm*mmHg	11,181 ± 2750	11,827 ± 1736	0.365
Increase in RPP, %	49 ± 18	38 ± 29	0.206
MPRI, ratio	2.01 ± 0.42	1.60 ± 0.44	0.008

Values are mean ± standard deviations or percentages. T2DM indicates type 2 diabetes mellitus; CMR, cardiovascularmagnetic resonance; bpm, beats per minute; MPRI, myocardial perfusion reserve index; RPP, rate pressure product

### Inter-study reproducibility, inter-observer and intra-observer variability of stress native T1 mapping

The inter-study reproducibility, inter-observer and intra-observer variability of mid-ventricular ShMOLLI T1-map stress reactivity were assessed.

5 healthy subjects (2 males, 3 females; age 41 ± 23 years; all 1.5 T, mid-ventricular slice stress/rest). All 10 scans anonymized and analyzed in random order. The interval between scans for these participants undergoing test-retest inter-study reproducibility was 3 ± 2 years apart. There was a good agreement between the first and second scans (0.33 ± 1.13%). Mean ΔT1 for the first scan (6.2 ± 1.0%) was not significantly different compared to the second scan (6.5 ± 1.0%), paired t-test *p* = 0.55.

There was a good agreement for inter-observer (−0.3 ± 1.7%) and intra-observer assessments (−0.2 ± 1.4%) by Bland-Altman plotting. Coefficient of determination showed strong relationship for inter-observer (r^2^ = 0.759) and intra-observer (r^2^ = 0.837) assessments.

### Changes in rest and adenosine stress T1 mapping

**Fig. 2:**
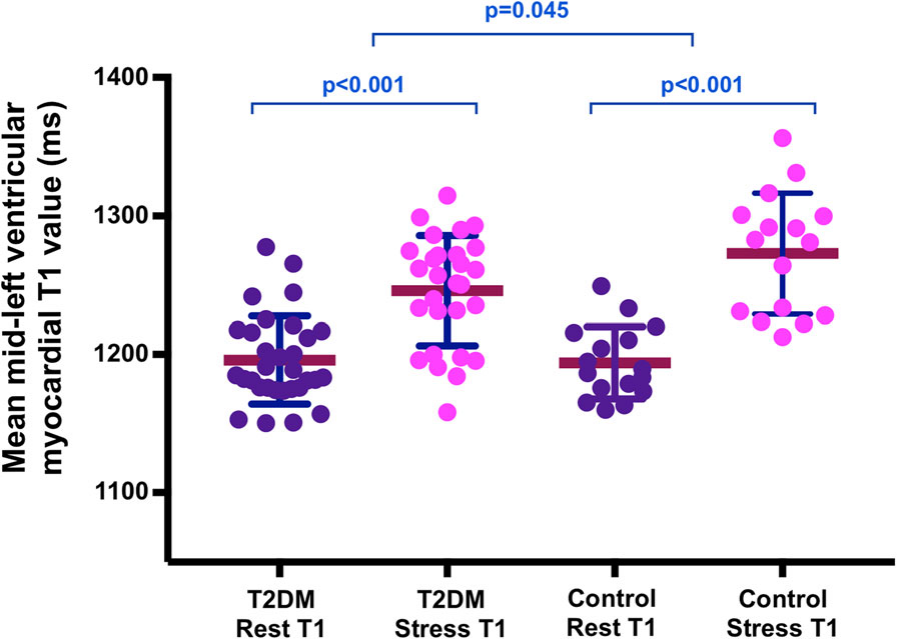
Differences in rest and adenosine stress LV myocardial T1 values between healthy controls and patients with T2DM. Scatter columns show mean LV myocardial T1 relaxation times and error bars indicate standard deviations. The lower *p*-values represent the differences in rest and adenosine stress LV myocardial T1, the upper *p*-value represent the represent the statistical difference in relative T1 reactivity between controls and patients with T2DM

**Fig. 3:**
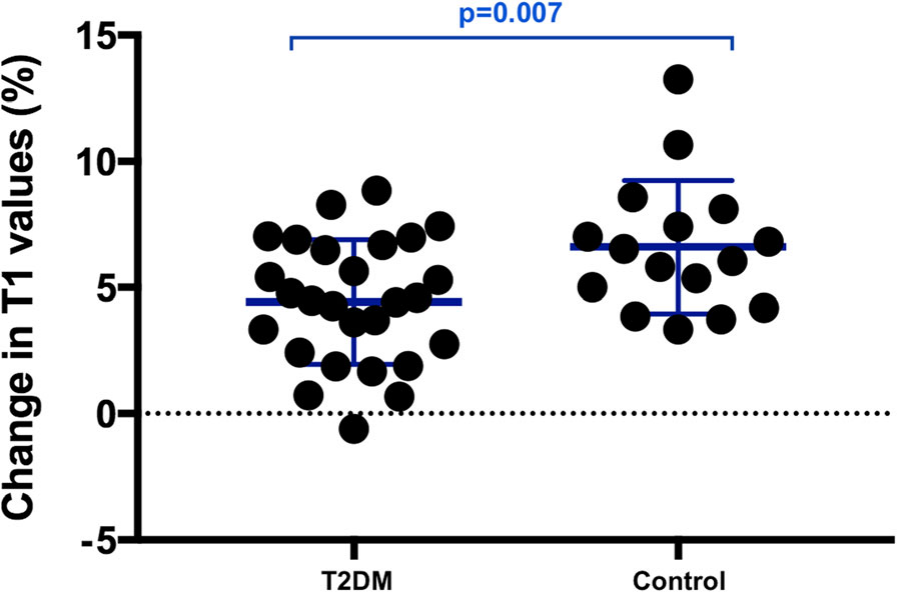
LV myocardial T1 reactivity to adenosine stress in patients with T2DM and healthy controls. Scatter columns show mean percentage change in T1 with adenosine stress and error bars indicate standard deviations

There were no significant differences in native myocardial non-contrast T1 values between T2DM patients and healthy controls at rest. During adenosine stress, T1 values increased significantly in both T2DM patients (from 1196 ± 32 ms to 1244 ± 44 ms, *P* < 0.001) and healthy controls (from 1194 ± 26 ms to 1273 ± 44 ms, *p* < 0.001) **(**Fig.2 **)**. However, stress T1 response was significantly blunted in T2DM patients compared to healthy controls (T2DM ΔT1 = 4.1 ± 2.9% vs. healthy controls ΔT1 = 6.6 ± 2.6%, *p* = 0.007) **(**Fig.3 **)**, as was the maximal T1 during adenosine stress (T2DM: 1244 ± 44 ms vs. healthy controls: 1273 ± 44 ms, *p* = 0.045). A sub-group analysis of lean T2DM patients (BMI < 25) vs lean controls for the ΔT1 was performed and our results showed in the absence of obesity or even overweight status T2D is associated with T1 (3.51 ± 2.18 vs lean controls 6.21 ± 1.72, *p* = 0.013).

There was no significant correlation between the percent change of T1 and MPRI. However, there was a significant correlation between the adenosine stress T1 and MPRI (*r* = 0.35, *p* = 0.031), when there was no significant correlation between rest T1 and MPRI (*r* = −0.19, *p* = 0.31).

Maximal T1 during adenosine stress and T1 reactivity (ΔT1) correlated negatively with LV mass index (*r* = −0.50, *p* = 0.001; *r* = −0.40, *p* = 0.018) and LV concentricity (LV mass to LV EDV ratio)(*r* = −0.33, *p* = 0.027; *r* = −0.41, *p* = 0.005) in the study group as a whole, but no significant correlation in separate groups.

### Extra-cellular volume (ECV) quantification

There was no significant difference in ECV between T2DM patients and controls (T2DM 30 ± 3%, vs healthy controls 30 ± 2%, *p* = 0.91).

## Discussion

In this study using CMR stress and rest T1-mapping, we demonstrate that T2DM is associated with blunted native T1 response under adenosine stress even in highly selected T2DM patients with stable disease and no other comorbidities. The blunted maximal stress T1 response in T2DM, as well as the impaired myocardial perfusion reserve index, are consistent with attenuated vascular reactivity and coronary microvascular dysfunction. Additionally, we show a negative correlation between stress T1 and concentric LV remodelling, suggesting a link between the subclinical abnormalities in coronary vasodilatory function and adverse cardiac remodeling in diabetes. However, the causality of this relationship needs to be investigated in future studies.

Coronary microvascular dysfunction in diabetes is likely to be a multifactorial phenomenon, related to changes in perivascular and interstitial fibrosis [31], myocardial hypertrophy [32], reduced capillary density, and autonomic neuropathy [33]. Importantly, coronary microvascular dysfunction is an early precursor of cardiovascular events and was shown to be associated with a 2.5% annual major adverse event rate that includes cardiovascular mortality, nonfatal myocardial infarction, nonfatal stroke, and heart failure even among patients without epicardial coronary artery stenosis [34]. Consequently, early identification of coronary microvascular disease may be beneficial in prognosis evaluation and patient stratification for optimal medical therapy [35].

Myocardial perfusion is influenced by multiple factors including metabolic demand, diastolic time, and driving blood pressure [36]. The functional responsiveness of the microcirculation can be influenced by increased heart rate, reduced diastolic time, decreased driving BP. An increase in systolic intramyocardial and ventricular pressures, as it would be expected to occur in LV concentric remodelling, can also adversely affect myocardial perfusion. In this study, although it remained within the normal limits, the resting heart rates were higher in patients with T2DM compared to healthy controls. Furthermore, patients with T2DM also demonstrated LV concentric remodeling. The higher rate pressure product at rest in our patients with T2DM would be expected to cause a higher resting perfusion, as a result may lead to lower MPRI; on the other hand the LV concentric remodeling would be expected to reduce the resting perfusion. The influence of these important factors on the coronary physiology is intricate, and should be considered while interpreting the myocardial perfusion abnormalities in patients with diabetes.

### CMR T1-mapping for the assessment of coronary vasodilatory function

Heart failure is the leading cause of mortality in patients with T2DM [5] and importantly coronary microvascular dysfunction is considered among the pathophysiological alterations in diabetes responsible for cardiac dysfunction [1]. In the context of the global epidemic of diabetes, coronary vascular reactivity, as assessed by rest and adenosine stress T1-mapping, may serve as an additional biomarker for disease severity and therapeutic response without the need for gadolinium-based contrast agents. Furthermore, this gadolinium-free technique is easily applicable, independent of heart rate and robust to tachyarrhythmias [14, 37], making it highly suitable for CMR scanning during vasodilator stress. This study shows that the technique is sensitive to detecting subtle changes in vascular reactivity in this patient group, in agreement with traditional first pass perfusion, paving the way to future mechanistic studies of further alterations of ΔT1 in overt diabetic cardiomyopathy, or for use in patients with diabetes and more significant comorbidities, including renal failure. CMR stress/rest T1 mapping may prove a convenient tool to assess potential beneficial effects of novel therapies designed for patients with T2DM on the myocardium and coronary vasculature, and to monitor drug response, without the need for repeated exposure to gadolinium-based contrast agents.

First-pass myocardial perfusion CMR during vasodilatory stress directly assesses reductions in microvascular blood flow with high diagnostic accuracy. CMR studies of the coronary circulation exploit the first-pass kinetics of T1-enhancing extracellular gadolinium-based contrast media. During the first pass, the contrast medium diffuses into the interstitial space from the microvasculature, resulting in an increase in signal intensity that is proportional to the perfusion and blood volume of the tissue, the extravascular compartment size, and capillary permeability.

It is conceivable that in the future adenosine rest/stress T1 mapping may become a valuable test in addition to conventional clinical information and perfusion assessment for patients with diabetes. Early identification of changes using these techniques may lead to improved clinical outcomes by preventive interventions. This method may provide surrogate markers of long-term prognostic effects without the need for gadolinium, making it also available for diabetic patients with end-stage kidney disease. However, reproducibility assessments, transferability between centers, and translatability between field strengths and vendors, are requirements which are not yet all fulfilled with adenosine stress and rest T1 mapping [38] and future studies are needed to address these lacunae, innovatively. As a result, first pass perfusion imaging persists as the robust and validated tool for assessment of myocardial perfusion, despite its limitations in patients without contraindications to use contrast agents.

### Study limitations

This study was limited by a relatively small sample size, and larger-scale future studies with long follow-up are necessary to determine the prognostic value of stress T1 response. Furthermore, as an observational study, we cannot determine the mechanisms for the observed differences in MPRI and T1 stress response.

Whilst the demonstration of a robust inter-study reproducibility for any technique is important to support the technique’s clinical and research application, due to ethical considerations regarding a second administration of adenosine with an additional hospital visit in this study was deemed too high a burden on study subjects as this could lead to higher risk of adverse event rates, and high drop-out rates. Consequently, in our study we have not assessed the inter-study reproducibility of the adenosine stress T1 mapping. Further studies are needed to demonstrate the inter-study reproducibility of adenosine stress T1 mapping.

Although resting heart rates were statistically higher in patients, we have previously demonstrated that T1 estimation using the ShMOLLI technique is independent of heart rate variations (40 to 100 beats/min) over the applicable range of T1 values in phantoms and simulations at 1.5- and 3.0-Tesla [39]. Therefore, any potential confounding effects due to technical heart rate dependencies on the findings in this study are negligible, and the observed T1 values most likely reflect true physiologic and pathophysiologic changes in healthy controls and patients, respectively.

Despite statistical differences in stress ΔT1 between T2DM patients (4.1 ± 2.9%) and healthy controls (6.6 ± 2.6%), there are numerical overlaps between these values. The effect size to detect significant differences between diabetic patients and healthy controls using stress ΔT1 is respectable (Cohen’s d 1.2), which is comparable to the difference in myocardial perfusion reserve index between fractional flow reserve -negative (MPRI 1.8 ± 0.5) and fractional flow reserve-positive (MPRI 1.2 ± 0.3) epicardial coronary lesions using gadolinium-enhanced stress perfusion CMR (Cohen’s d 1.4 [40]). Liu et al., previously showed that stress T1-mapping is independent of field-strength between 1.5 and 3 T [18]. Considering the abolished stress ΔT1 values for ischemic myocardium (0.2 ± 0.8%) at 1.5 T [41], the effect size for detecting a significant difference between diabetic patients (4.1 ± 2.9%) vs. ischemic myocardium (0.2 ± 0.8%) is higher (Cohen’s d 1.8). This suggests that the detection of myocardial ischemia in diabetic patients using gadolinium-free stress T1-mapping is possible, without the need for gadolinium contrast agents, and deserves further investigation.

Adenosine is the most commonly used agent for CMR perfusion studies, and it increases blood flow mostly by non-endothelial-dependent mechanisms via receptors on microvascular smooth muscle cells that modulate intracellular calcium [42]. Thus, the lower MPRIs and T1 stress responses observed in T2DM patients in this study might be the result of decreased vasodilation via both endothelium-dependent and -independent pathways. However, a complete characterisation of coronary microvascular function also requires the assessment of response to vasoconstrictor stimuli (acethylcholine, ergonovine), which can be performed only during an invasive coronary angiogram. Subjecting our participants to invasive coronary angiography, with even a small risk of complications, was deemed unacceptable for asymptomatic patients. Adenosine is shown to assess mostly non-endothelial-dependent coronary flow changes, and therefore we cannot completely rule out that endothelial-dependent coronary flow abnormalities may have affected our findings. Similarly, because we haven’t carried out invasive assessment of coronary physiology, the diameter changes in response to an exogenous donor of nitric oxide, isosorbide dinitrate, could not be assessed, and therefore impaired responsiveness of underlying vascular smooth muscle cells or structural changes of the vascular wall cannot be excluded with certainty.

Absolute quantification of myocardial perfusion would have been interesting for direct comparison with absolute T1 quantification under stress and rest conditions. However, quantitative perfusion analysis is relatively cumbersome, requiring specialized computational software with mathematical assumptions and some recognized limitations [43].

The incidence of T2DM is driven predominantly by the obesity epidemic, and there is no doubt that obesity is a strong contributor to diabetic cardiomyopathy [44]. There is a significant overlap between the obesity related and diabetes related heart disease phenotypes. Both conditions are associated with LV concentric remodeling [4, 45–47], coronary microvascular dysfunction, myocardial energetic impairment [44] and increased risk of heart failure [4]. In our study, a third of (11 out of 31) T2DM patients were obese (35 > BMI > 30), 10 out of 31 patients were normal weight (BMI < 25), and the remaining 10 patients were overweight (25 < BMI < 30). This study aimed to prove the concept that adenosine rest and stress non-contrast T1 mapping would detect significant differences in patients with T2DM free of obstructive epicardial CAD on CTCA, in-keeping with coronary microvascular dysfunction. In sub-group analysis we have detected significant differences in MPRI and T1reactivity, suggesting that in the absence of obesity or even overweight status T2D is associated with coronary microvascular dysfunction. Given the comorbidity of obesity in a third of our patients with T2DM in this study, impact of obesity on the cardiac abnormalities cannot be dismissed; however, this is in fact not within the scope of our study aims.

Finally, CCTA was not performed in the healthy cohort to prevent unnecessary ionizing radiation exposure. Significant CAD was deemed to be very unlikely in this cohort.

## Conclusions

Patients with well controlled T2DM, even in the absence of arterial hypertension and significant epicardial CAD, exhibit blunted maximal T1 response during adenosine vasodilatory stress, likely reflecting coronary microvascular dysfunction, as well as early signs of LV concentric remodelling without overt LVH. Adenosine stress and rest T1 mapping can detect subclinical functional abnormalities of the coronary microvasculature, without the need for gadolinium contrast agents.

In the future, CMR and particularly T1 mapping at stress may be used to identify early features of diabetic heart disease and subclinical risk markers in patients with T2DM, providing an opportunity for early therapeutic intervention.

## Abbreviations

BMI: Body mass index
BP: Blood pressure
bSSFP: Balanced steady state free precession
CAD: Coronary artery disease
CCTA: Coronary computed tomographic angiography
CMR: Cardiovascular magnetic resonance
ECG: Electrocardiogram
ECV: Extracellular volume fraction
LGE: Late gadolinium enhancement
LV: Left ventricle/left ventricular
MPRI: Myocardial perfusion reserve index
RPP: Rate pressure product
ShMOLLI: Shortened modified Look-Locker inversion recovery
T2DM: Type 2 diabetes mellitus
ΔT1: Change in myocardial T1 values from rest to vasodilator stress
