# Correction to: Adenosine stress CMR T1-mapping detects early microvascular dysfunction in patients with type 2 diabetes mellitus without obstructive coronary artery disease

**DOI:** 10.1186/s12968-017-0406-y

**Published:** 2017-12-07

**Authors:** Eylem Levelt, Stefan K. Piechnik, Alexander Liu, Rohan S. Wijesurendra, Masliza Mahmod, Rina Ariga, Jane M. Francis, Andreas Greiser, Kieran Clarke, Stefan Neubauer, Vanessa M. Ferreira, Theodoros D. Karamitsos

**Affiliations:** 1 University of Oxford Centre for Clinical Magnetic Resonance Research, Division of Cardiovascular Medicine, Radcliffe Department of Medicine, University of Oxford, Oxford, UK; 2 Department of Cardiovascular Sciences, University of Leicester, Leicester, UK; 3 Siemens Healthcare GmbH, Erlangen, Germany; 4 Department of Physiology, Anatomy and Genetics, University of Oxford, Oxford, UK; 5 1st Department of Cardiology, Aristotle University of Thessaloniki, AHEPA Hospital St. Kyriakidi 1, 54636 Thessaloniki, Greece

## Correction to: J Cardiovasc Magn Reson (2017) 19: 81. DOI: 10.1186/s12968-017-0397-8

In the original publication of this article [1] Fig. 1 was incorrect due to the use of a colour bar with wrong range in error. This is now corrected (Fig. 2 in the erratum).

**Fig. 1:**
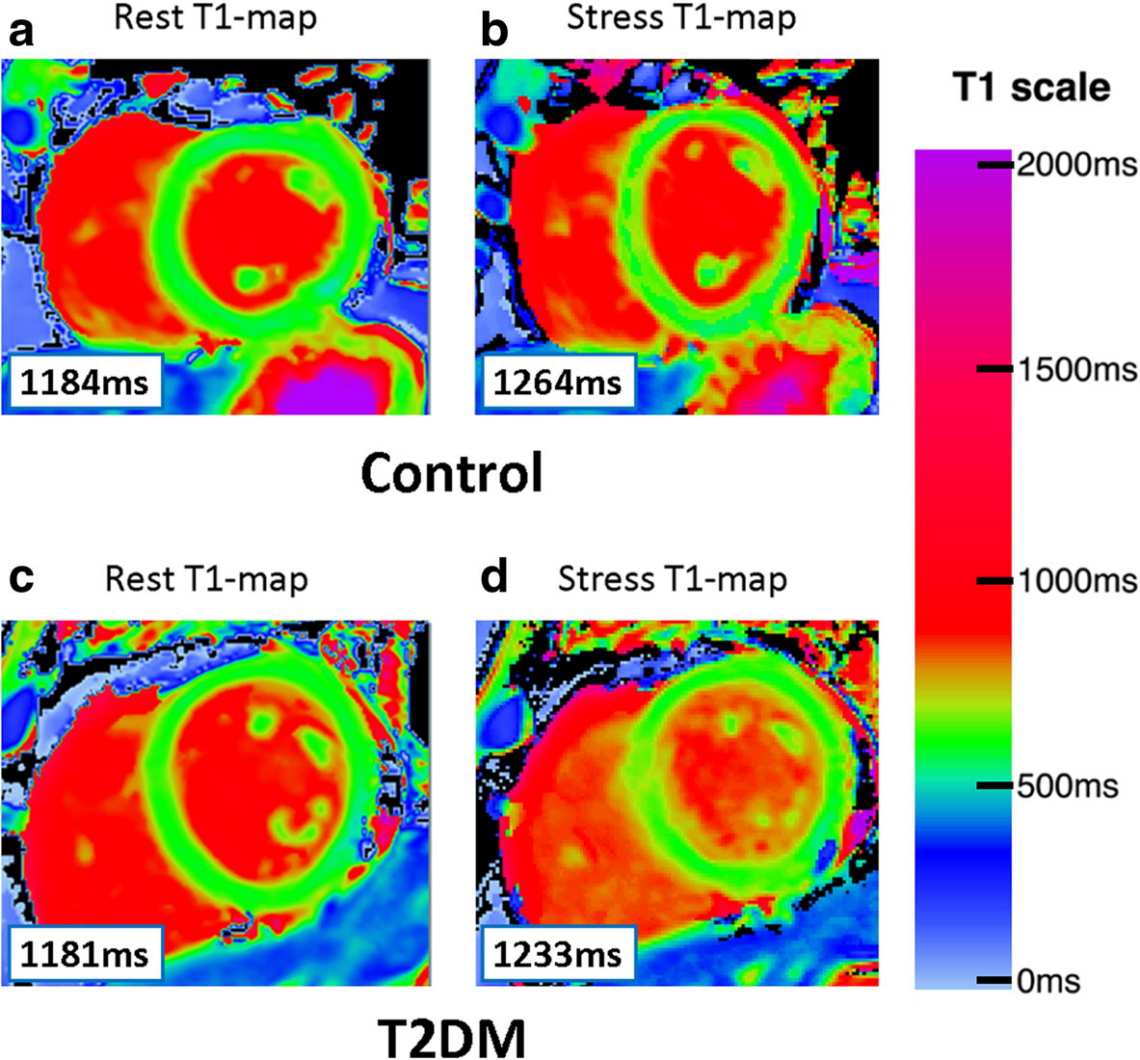
Original version of Fig. 1 as published on 25 October 2017

**Fig. 2:**
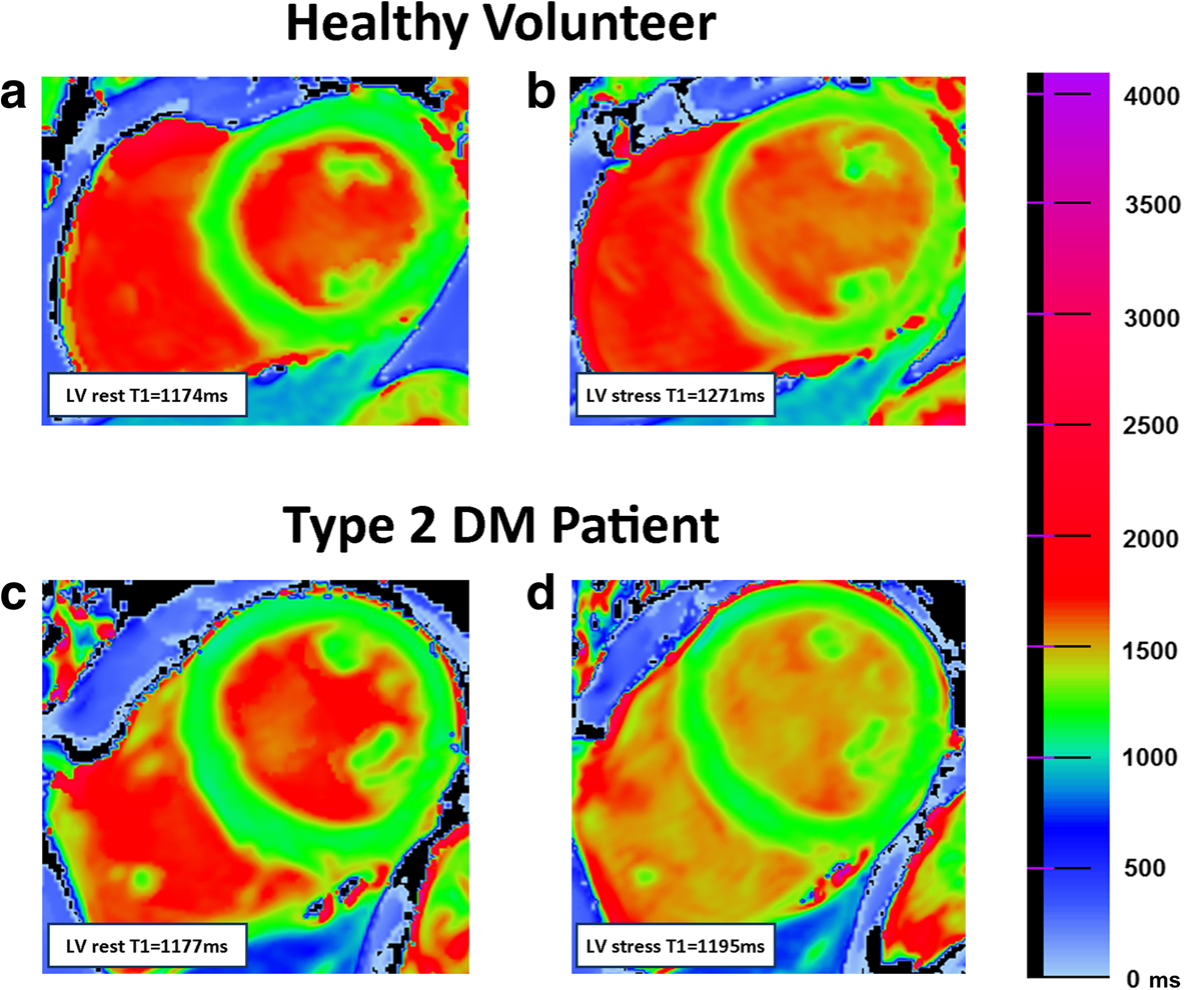
Corrected version of Fig. 1

## References

[1] Levelt E, Piechnik SK, Liu A (2017). Adenosine stress CMR T1-mapping detects early microvascular dysfunction in patients with type 2 diabetes mellitus without obstructive coronary artery disease. J Cardiovasc Magn Reson.

